# *Trypanosoma cruzi* Exploits Wnt Signaling Pathway to Promote Its Intracellular Replication in Macrophages

**DOI:** 10.3389/fimmu.2018.00859

**Published:** 2018-04-23

**Authors:** Ximena Volpini, Laura F. Ambrosio, Laura Fozzatti, Constanza Insfran, Cinthia C. Stempin, Laura Cervi, Claudia Cristina Motran

**Affiliations:** ^1^Departamento de Bioquímica Clínica, Facultad de Ciencias Químicas, Universidad Nacional de Córdoba, Córdoba, Argentina; ^2^Centro de Investigaciones en Bioquímica Clínica e Inmunología (CIBICI), CONICET, Haya de la Torre y Medina Allende, Ciudad Universitaria, Córdoba, Argentina

**Keywords:** *Trypanosoma cruzi* infection, Wnt proteins, beta-catenin, macrophages, cytokines, indoleamine 2,3-dioxygenase

## Abstract

During the acute phase of *Trypanosoma cruzi* infection, macrophages can act as host cells for the parasites as well as effector cells in the early anti-parasitic immune response. Thus, the targeting of specific signaling pathways could modulate macrophages response to restrict parasite replication and instruct an appropriate adaptive response. Recently, it has become evident that Wnt signaling has immunomodulatory functions during inflammation and infection. Here, we tested the hypothesis that during *T. cruzi* infection, the activation of Wnt signaling pathway in macrophages plays a role in modulating the inflammatory/tolerogenic response and therefore regulating the control of parasite replication. In this report, we show that early after *T. cruzi* infection of bone marrow-derived macrophages (BMM), β-catenin was activated and Wnt3a, Wnt5a, and some Frizzled receptors as well as Wnt/β-catenin pathway’s target genes were upregulated, with Wnt proteins signaling sustaining the activation of Wnt/β-catenin pathway and then activating the Wnt/Ca^+2^ pathway. Wnt signaling pathway activation was critical to sustain the parasite’s replication in BMM; since the treatments with specific inhibitors of β-catenin transcriptional activation or Wnt proteins secretion limited the parasite replication. Mechanistically, inhibition of Wnt signaling pathway armed BMM to fight against *T. cruzi* by inducing the production of pro-inflammatory cytokines and indoleamine 2,3-dioxygenase activity and by downregulating arginase activity. Likewise, *in vivo* pharmacological inhibition of the Wnts’ interaction with its receptors controlled the parasite replication and improved the survival of lethally infected mice. It is well established that *T. cruzi* infection activates a plethora of signaling pathways that ultimately regulate immune mediators to determine the modulation of a defined set of effector functions in macrophages. In this study, we have revealed a new signaling pathway that is activated by the interaction between protozoan parasites and host innate immunity, establishing a new conceptual framework for the development of new therapies.

## Introduction

Chagas’ disease, caused by the protozoan parasite *Trypanosoma cruzi*, represents a major cause of heart disease and cardiovascular-related deaths in endemic areas and causes a significant economic burden on the affected countries. Approximately 8 million people are infected with *T. cruzi* in Central and South America, and at least 120 million are at risk of infection (1). Currently, there are no vaccines available to prevent Chagas disease, and treatment options are limited to anti-parasitic drugs that are expensive, not well tolerated, and effective only during short periods of the acute phase (2).

The entry of metacyclic trypomastigotes (Tps) of *T. cruzi* into the mammalian host initiates the invasion by these parasites of several host cell types where they are converted into amastigotes, which are the replicative form of this parasite (3). During the acute phase of the infection macrophages represent an important target of *Trypanosoma cruzi* and therefore, these cells are central for the control of this pathogen. Within macrophages, the replication of *T. cruzi* can be either inhibited or favored leading to the dissemination of the parasite (4). Thus, it has been reported that during the early phase of infection, the control *T. cruzi* parasitism is dependent on macrophage activation through toll-like receptors (TLRs) and their subsequent activation by pro-inflammatory cytokines. Activated macrophages upregulate inducible nitric oxide synthase (iNOS) and indoleamine 2,3-dioxygenase (IDO) enzymes, leading to the production of nitric oxide (NO) and kynurenines, with both being important effector molecules that induce death of the amastigotes (5–9). The protective mechanisms of cell-mediated immunity (Th1 cells) are required for the resistance during this infection; nevertheless, unbalanced Th1 cells can also orchestrate a deleterious response that can cause tissue damage and fibrosis, since high levels of NO, IFN-γ, and tumor necrosis factor (TNF) have been associated with the pathogenesis of chronic Chagas disease (10–14). Therefore, a better understanding of the cellular and molecular mechanisms that orchestrate the different signals that promote the effective macrophage activation (able to restrict parasite replication) followed by its opportune contraction to prevent immunopathology is mandatory to design improved therapeutic strategies.

The Wnt pathway is an evolutionarily highly conserved signaling system that plays a critical role in cellular development, motility, polarization, survival, and proliferation (15, 16). In the last years, emerging studies have highlighted that the Wnt signaling pathway, particularly in dendritic cells (DC) and macrophages, plays a major role in regulating tolerance versus immunity. Members of the Wnt family are lipid-modified glycoproteins secreted by different cell types that bind to G-protein-coupled receptors of the Frizzled (Fzd) family and different coreceptors to activate a signaling cascade involved in complex mechanisms of gene regulation. In mice and human, 19 ligands secreted glycoproteins of the Wnt family, 10 seven-transmembrane receptors of the Fzd family, as well as several coreceptors or alternative receptors are known (16, 17). Depending on the composition of the Wnt–Fzd-coreceptor complex and the cellular context, Wnt proteins can initiate at least three different intracellular signaling cascades that regulate many cellular events: the Wnt/β-catenin called canonical pathway, the Wnt/Planar cell polarity pathway, and the Wnt/Ca^+2^ pathway (18, 19). In resting state, cytosolic/nuclear β-catenin is maintained at a very low level through rapid turnover of free β-catenin. This turnover is executed through a multi-protein complex, termed the β-catenin destruction complex, integrated by AXIN1/2, adenomatous polyposis coli, casein kinase I-alpha, and glycogen synthase kinase 3 beta (GSK-3β). GSK-3β sequentially phosphorylates β-catenin with β-catenin hyperphosphorylated subjected to ubiquitination and proteasomal degradation. Activation of Wnt/β-catenin signaling pathway results in the inhibition of the activity of GSK-3β, which leads to the accumulation of β-catenin in the cytoplasm and its translocation to the nucleus, where it interacts with T-cell factor/lymphoid enhancer factor family members and regulates the transcription of several target genes (16, 17). While canonical Wnt signaling has been extensively studied from the view point of innate and adaptive immune response, the non-canonical Wnt/Ca^+2^ signaling cascade has been less focused on. The binding of Wnt ligand to cognate Fzd receptor leads to the activation of calcium/calmodulin-dependent kinase II (CaMKII) and protein kinase C (PKC). Both CaMKII and PKC activate many nuclear transcription factors as NF-κB and CREB. Similarly, calcium ions can activate widely expressed protein phosphatase calcineurin that can activate cytoplasmic protein nuclear factor associated with T cells (NFAT) *via* dephosphorylation. This event results in accumulation, nuclear translocation and transcription of several NFAT target genes, including pro-inflammatory genes in lymphocytes (20).

Human and murine DC and macrophages express various receptors and Wnt proteins and are susceptible to both canonical and non-canonical signaling, and recently, several studies have emphasized the important role of DC and macrophages Wnt/β-catenin pathway in regulating inflammatory responses to microbial infections (21, 22). In DC, the activation of canonical and Wnt/Ca^+2^ pathways is associated with the development of a tolerogenic profile, secretion of anti-inflammatory cytokines, induction of Treg cells, and the inhibition of the adaptive immune response (23–28). However, Wnt pathways activation in macrophages may have both pro- and anti-inflammatory consequences, and different Wnt proteins may effectively cross-regulate each other in macrophages [reviewed in Ref. (21, 29)]. Wnt proteins and Fzd receptors are induced by STAT3-, TLR2-, TLR4-, and NF-κB-mediated signaling and, in addition to direct activation of β-catenin by Wnt proteins, TLR-mediated signals can also directly activate β-catenin (21, 26, 28, 30–37). Considering that TLR2 and TLR4 are involved in the recognition of different *T. cruzi* components (38–40), here we tested the hypothesis that during *T. cruzi* infection, the activation of Wnt signaling pathway in macrophages plays a role modulating the inflammatory/tolerogenic response and therefore regulating the control of intracellular parasite replication.

## Materials and Methods

### Mice and Parasites

All animal experiments were approved by and conducted in accordance with guidelines of the Animal Care and Use Committee of the Facultad de Ciencias Químicas, Universidad Nacional de Córdoba (Approval Number HCD 831/15). C57BL/6 (B6) was obtained from School of Veterinary, La Plata National University (La Plata, Argentina). All animals were housed in the Animal Facility of the Facultad de Ciencias Químicas, Universidad Nacional de Córdoba (OLAW Assurance number A5802-01). The Tulahuen strain of *T. cruzi* was used, which was maintained by weekly i.p. inoculations in mice.

### Reagents

Macrophages were cultured in complete medium: RPMI 1640 (Gibco, ThermoFisher Scientific) supplemented with 2 mM GlutaMAX (Gibco, ThermoFisher Scientific), 10% heat-inactivated fetal calf serum (Gibco, Thermo Fisher), and 50 µg/mL Gentamicin (Gibco, ThermoFisher Scientific). PBS was purchased from Gibco (ThermoFisher Scientific); bovine serum albumin and DMSO were obtained from Sigma-Aldrich.

### Cells

Bone marrow-derived macrophages (BMM) were generated as described previously (41). Peritoneal macrophages (PM) were obtained as described previously (42). J774 murine macrophages and the human monocytic THP-1 cell lines were obtained from the American Type Culture Collection (Manassas, VA, USA). THP-1 cell line was differentiated into macrophages by culture in complete medium supplemented with of 50 nM phorbol 12-myristate 13-acetate (Sigma-Aldrich) for 24 h followed by 24 h of incubation in complete medium.

### *In Vitro* Infections and Treatments

Mo were incubated with LiCl (10 mM, #746460 Sigma-Aldrich), BIO (5 µM, #3194 TOCRIS), iCRT14 (50 µM, #4299, TOCRIS), CCT036477 (20 µM, #BML-WN114, ENZO), IWP-L6 (20 µM, #504819, Calbiochem), IFN-γ (10 ng/mL, Sigma-Aldrich) plus LPS (10 µg/mL, from *Escherichia coli* serotype 0111:B4, Sigma-Aldrich), 1-methyl-d-tryptophan (1-MT, #860646, Sigma-Aldrich) or complete medium before being infected with *T. cruzi* Tps (Mo:Tps = 1:3). Culture medium with PBS or DMSO was used as vehicle control for LiCl and 1-MT or iCRT14, CCT036477, and IWP-L6, respectively. MeBIO (5 µM, #3873, TOCRIS) was used as control of BIO.

### *In Vitro* Trypanocidal Activity Assay

The growth of parasites in Mos was evaluated by counting the intracellular amastigotes by immunofluorescence assay using serum of patient with chronic Chagas disease as described previously (8). The number of intracellular amastigotes was calculated by dividing intracellular parasites (*n*/200 cells) in differentially treated cultures by number of intracellular parasites (*n*/200 cells) in the corresponding vehicle-treated cultures, and expressed as relative units.

### Western Blotting

Spleen cells or BMM treated with RIPA buffer were analyzed by 10% SDS-PAGE and transferred to a nitrocellulose membrane. After blocking with 5% milk the membranes were incubated with the following antibodies: anti-β-catenin (15B8, eBioscience), anti-p-Ser552-β-catenin (D8E11, Cell Signaling), anti-NFATc1 (7A6, Santa Cruz Biotechnology), anti-p-Thr286-CaMKII (D21E4, Cell Signaling), anti-p-Ser9-GSK-3β (37F11, Cell Signaling), anti-Wnt3a (ab19925-100 polyclonal, Abcam), anti-Wnt5a (ab72583 polyclonal, Abcam), anti-IDO antibody (Santa Cruz Biotechnology) followed by anti-rabbit or -mouse fluor-coupled secondary antibody. Anti-β-actin (mAbcam8226, Abcam) was used as loading control. The blots were revealed by incubation with corresponding IRD Fluor 800-labeled IgG or IRD Fluor 680-labeled IgG secondary antibody (LI-COR Inc., Lincoln, NE, USA) for 1 h at room temperature. After washing, the membranes were scanned with the Odyssey infrared imaging system (LI-COR, Lincoln, NE, USA) at a wavelength of 700–800 nm. Densitometric analysis was performed using ImageJ software.

### β-Catenin and NFATc1 Immunofluorescence

The cells were fixed in 4% formaldehyde and perm with 10% TritonX-100 for 15 min, washed three times with 0.01 M PBS, and incubated with 5% bovine serum albumin (Sigma-Aldrich) for 1 h for blocking. The sections were then incubated overnight at 4°C with anti-β-catenin (15B8, eBioscience) or anti-NFATc1 (7A6, Santa Cruz Biotechnology) primary antibodies. Next, the cells were washed three times with 0.01 M PBS, incubated with secondary antibody Alexa Fluor 488-conjugated Goat anti-Mouse IgG (#A11001, Invitrogen) for 4 h and washed three times with 0.01 M PBS. For nuclear counterstaining, the cells were incubated with 4′,6-diamidino-2-phenylindone (Cell Signaling Technology, #4083) for 10 min, washed in 1× PBS, and the slides were set with FluorSave™ reagent (EMD Millipore). Expression and localization of beta-catenin were observed with an Olympus FV1200 laser scanning confocal microscope (Olympus Corporation) with fixed exposure time for all samples. Colocalization was quantified as a ratio of overlapping pixels to the total number of pixels by Threshold Mander’s coefficient and expressed as nuclear percentage (% nuclear). The preparations were visualized using confocal microscopy (Olympus FV1200), and the analyses of images were carried out using FIJI/ImageJ.

### Measurement of Cytokine Production

Cytokines were measured in culture supernatants of BMM 24 h post-infection (pi) by capture ELISA using antibodies and protocols suggested by the manufacturer (eBioscience). The cytokine concentration was expressed as index of cytokine release (Index) obtained by dividing the cytokine release of inhibitors/activators-treated BMM by the cytokine release of vehicle-treated BMM.

### Quantitative Real-Time-PCR (q-PCR)

RNA was extracted from BMM by the Trizol reagent (Invitrogen, ThermoFisher Scientific) and reverse-transcribed into cDNA by using Revert Aid First Strand cDNA Synthesis (Fermentas). Transcripts were quantified by real-time quantitative PCR on an StepOnePlus™ Real-Time System (ThermoFisher Scientific) using SYBR Green (ThermoFisher Scientific) with the following primers (all primers listed in the 5′ to 3′ orientation): *Wnt3a* TTC TTA CTT GAG GGC GGA GA (forward) and CTG TCG GGT CAA GAG AGG AG (reverse); *Wnt5a* GCA GGA CTT TCT CAA GGA CA (forward) and CCC TGC CAA AGA CAG AAG TA (reverse); *Ctnnb1* AGC CGA GAT GGC CCA GAA T (forward) and AAG GGC AAG GTT TCG AAT CAA (reverse); *Ccnd1* AGT GCG TGC AGA AGG AGA TT (forward) and CAC AAC TTC TCG GCA GTC AA (reverse); *Axin1* CTG GGC TTG TAT CCC ACT GT (forward) and ACC AAG CTG GTG GCT AGA GA (reverse); *Wisp1* CTG GAC AGA AAA GGG CAT GT (forward) and AGG AAG GAG GGG AAA TCT CA (reverse); *Fzd4* CTG CAG CAT GCC TAA TGA GA (forward) and CGT CTG CCT AGA TGC AAT CA (reverse); *Fzd6* TCC GAC GCT TGA AGA AAA CT (forward) and CAA CCC CAG GTC CTC AAG TA (reverse); *Fzd8* GCA GCA TGT TCG CTA TGA AA (forward) and AGT AGC CTG CTA TGG CCT CA (reverse); *Fzd9* AGA GCC TGT GCT ACC GAA AA (forward) and CAA GGA GGG AGC AAC CAT AA (reverse); *Actb* CGC CAC CAG TTC GCC ATG GA (forward) and TAC AGC CCG GGG AGC ATC GT (reverse). Relative expression was normalized to β-actin (*Actb*) and expressed as mRNA relative levels. The cycling conditions included a hot start at 95°C for 10 min, followed by 40 cycles at 95°C for 15 s and 60°C for 1 min. Specificity was verified by melting curve analysis and agarose gel electrophoresis.

### Arginase and IDO Activity

Arginase and IDO assays were performed as described previously (8, 43) with cell lysates that had been cultured in different conditions as indicated in the figure legends.

### NO Assay

The production of NO was measured indirectly by assaying nitrites in the culture supernatant using the Griess reaction as described previously (8).

### Lactate Dehydrogenase (LDH) Assay

Lactate dehydrogenase release was measured in the supernatant of BMM using LDH colorimetric assay kit (Wiener Lab) following the manufacturer’s protocol and expressed as international units (I/U).

### IWP-L6 Treatment

Groups of 6 mice (6–8 weeks old) maintained under standard conditions were infected with 5,000 bloodstream *T. cruzi* Tps by the i.p. route. On days 5, 8, 11, and 14 pi, mice were treated with IWP-L6 (7.5 mg/kg/dose) by the i.p. route. We resuspended IWP-L6 in DMSO (10 mg/mL) and then injected diluted in PBS, with this vehicle also being employed as a negative control. The levels of parasitemia were monitored every 2 days as described earlier, and the number of deaths was recorded daily. Determination of tissue parasitism was assessed in heart and liver obtained from 180-day-infected IWP-L6 or control mice as described previously (7).

### Statistical Analysis

Statistical analyses were performed using Student’s *t*-test, and two-way ANOVA followed by Bonferroni’s post-test for comparing more than two groups. The Kaplan–Meier analysis test was used for comparing survival of control and treated mice. Percent survival at each time was also compared by using the log-rank and Gehan–Breslow–Wilcoxon tests. The results were considered significantly different when *P* < 0.05.

## Results

### *T. cruzi* Infection Promotes Wnt Signaling Pathway Activation in Spleen Cells

*Trypanosoma cruzi-*infected B6 mice have great difficulty in controlling inflammatory response, resulting in the premature death of these animals by liver failure. Interestingly, we have observed that B6, unlike BALB/c mice, did not expand the population of Treg cells in parallel with the large expansion undergone by the T cell compartment resulting in an increased ratio of T effector/Treg cells (not shown). These results suggest that, as observed in patients with severe chronic Chagas disease cardiomyopathy (12, 44), the fatal outcome in B6 mice may be linked to an unbalanced inflammatory response. For that, although in *T. cruzi*-infected B6 mice the main target organ of pathological inflammatory response is the liver, and not the heart as in infected human patients, we performed the experiments in B6 mice to evaluate both parasite replication and inflammatory pathology.

**Figure 1:**
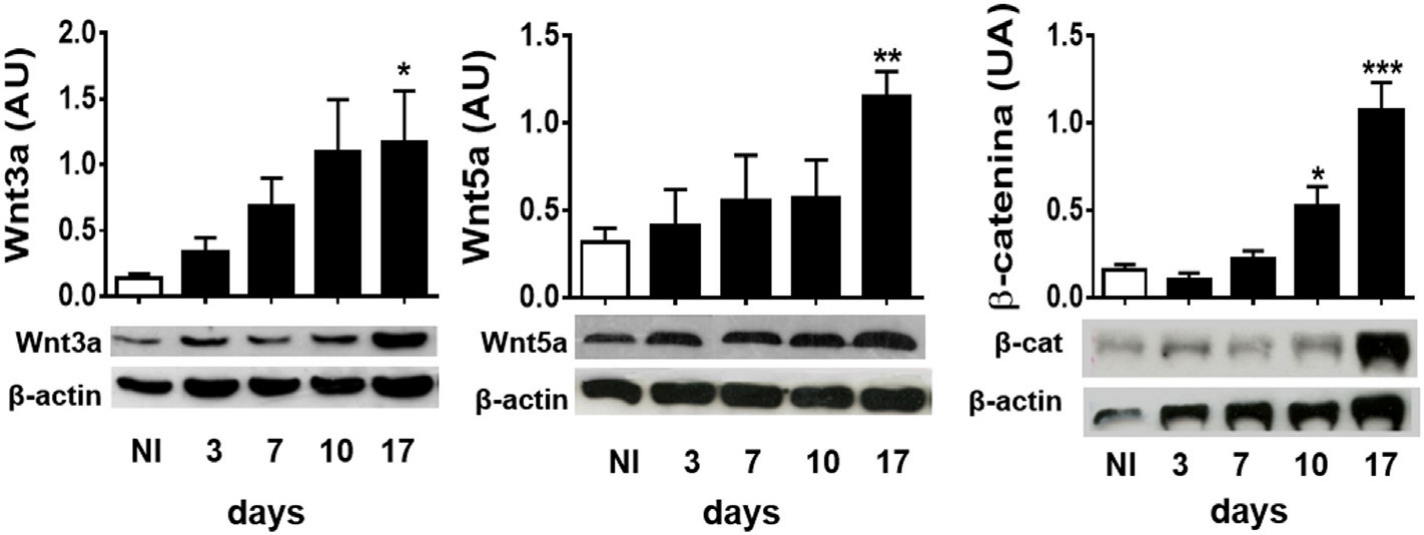
Experimental *Trypanosoma cruzi* infection induces the expression of Wnt pathway proteins. Western blot analysis of Wnt3a, Wnt5a, and β-catenin expression in spleen mononuclear cell homogenates derived from uninfected (NI) or *T. cruzi*-infected B6 mice at different times post-infection. Images show one representative NI and one infected mice by time point. Each bar represents the mean ± SEM of protein relative expression levels quantified by scanning the intensity of bands areas in the homogenates normalized to β-actin (*n* = 5 mice per time point) (*P < 0.05; **P < 0.01; and ***P < 0.001).

To study whether experimental infection with *T. cruzi* induces the production of Wnt pathway proteins, the expression of the most common inflammation-linked Wnt proteins such as Wnt3a and Wnt5a, and β-catenin was evaluated in spleen cells from infected B6 mice at different times pi. Figure 1 shows that, as *T. cruzi* infection progresses, the expression of Wnt3a, Wnt5a, and β-catenin is positively regulated in spleen cells. These results suggest that *T. cruzi* recognition induces in spleen cells the expression of Wnt proteins that could signal for the activation of the Wnt/β catenin canonical pathway, taking into account the accumulation of β-catenin.

### *T. cruzi* Infection Induces Expression of Wnt Proteins and Fzd Receptors in Macrophages

**Figure 2:**
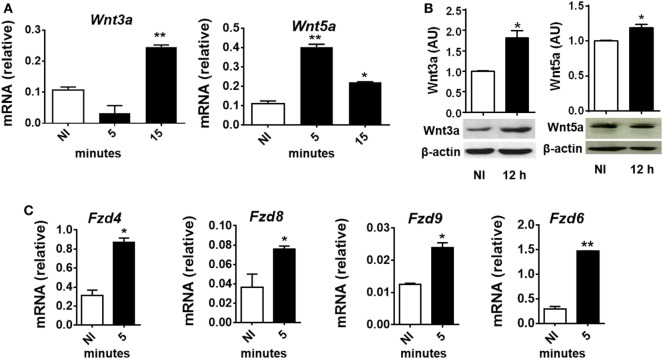
*In vitro Trypanosoma cruzi* infection induces expression of Wnt proteins, Frizzled (Fzd) receptors, and β-catenin macrophages. Bone marrow-derived macrophages were *in vitro* infected with *T. cruzi* trypomastigotes or left uninfected (NI) and then evaluated for expression of Wnt proteins and Fzd receptors at different times post-infection (pi). **(A)** Expression of Wnt3a and Wnt5a mRNA (relative to β-actin) was determined by quantitative real-time-PCR. **(B)** The relative abundance of Wnt3, Wnt5a, and β-actin in the cell lysates were determined by Western blot and densitometry at 12 h pi. Representative Western blot and the ratio of Wnt3a or Wnt5a to β-actin are shown. **(C)** Expression of Fzd4, Fzd8, Fzd9, and Fzd6 mRNA (relative to β-actin) was determined by q-PCR. The results are expressed as the average of three independent experiments ± SEM. Abbreviation: AU, arbitrary units (*P < 0.05 and **P < 0.01).

To evaluate whether *T. cruzi* experimental infection induces the expression of proteins involved in Wnt signaling in macrophages, BMM were *in vitro* infected with *T. cruzi* Tps and the expression of Wnt3a and Wnt5a transcripts and proteins determined by q-PCR and Western blot. Figures 2A,B show that *in vitro* infection of BMM with *T. cruzi* induced the expression of Wnt5a and Wnt3a transcripts and proteins. The evaluation by means of q-PCR of the transcripts corresponding to Fzd receptors that most strongly interact with Wnt3a and Wnt5a, such as Fzd4, Fzd6, Fzd8, and Fzd9 (45), revealed that as early as 5 min pi, there is an increase in the transcription of these genes (Figure 2C), suggesting that after the recognition of *T. cruzi* by innate immune receptors start the transcription of Wnt proteins that could signal through Fzd receptors to activate the Wnt signaling pathways.

### *T. cruzi* Infection Induces First β-Catenin Activation and Then Activates Wnt/Ca^+2^ Pathway in Macrophages

**Figure 3:**
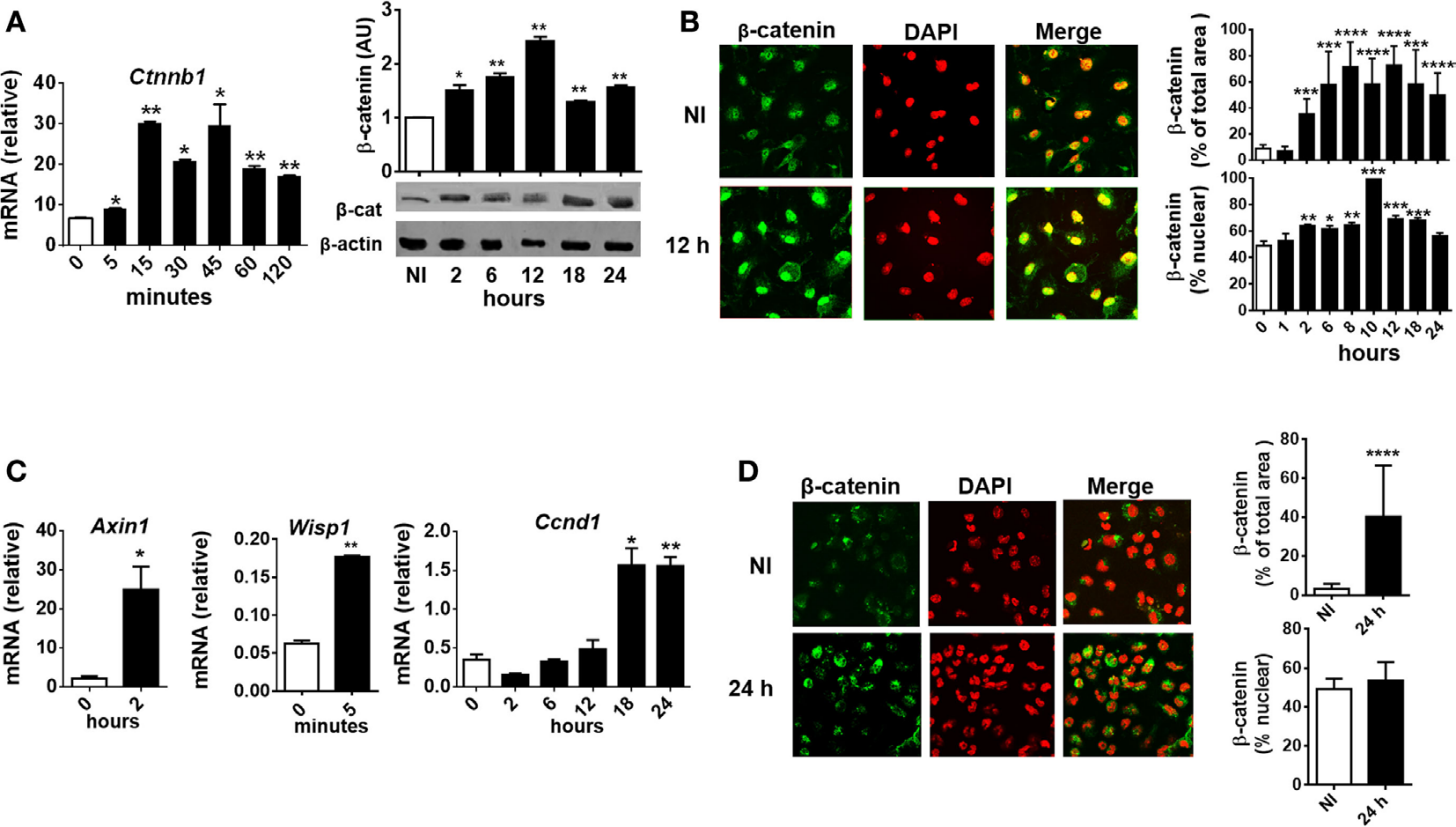
*Trypanosoma cruzi* infection induces early β-catenin activation in macrophages. Bone marrow-derived macrophages were *in vitro* infected with trypomastigotes of *T. cruzi* or left uninfected (NI) and then evaluated for β-catenin activation. **(A)** mRNA and protein expression levels of β-catenin by q-PCR and Western blot at different times post-infection (pi). β-Catenin mRNA relative to β-actin is shown. A representative Western blot and the ratio of β-catenin to β-actin are shown. The results are expressed as the average of three independent experiments ± SEM. Abbreviation: AU, arbitrary units. **(B)** Expression and localization of β-catenin by immunofluorescence and confocal microscopy. **(C)** mRNA relative expression of β-catenin target genes Axin1, Wisp1, and Ccnd1 by q-PCR. mRNA expression levels normalized over the expression of β-actin are expressed as the average of three independent experiments ± SEM. **(D)** Peritoneal macrophages were obtained from uninfected or infected mice at 24 h pi, and the levels of expression and localization of β-catenin were evaluated by immunofluorescence and confocal microscopy. In panels **(B,D)**, a representative field for each group is shown [**(B,D)**, 1,200×]. Nuclear staining was detected with 4′,6-diamidino-2-phenylindone (DAPI), and the levels of expression of β-catenin (% of total area) and the threshold Mander’s colocalization (% nuclear) coefficients calculated using FIJI/ImageJ program as described in Section “Materials and Methods.” Green, β-catenin; red, DAPI (*P < 0.05; **P < 0.01; ***P < 0.001; and ****P < 0.0001).

To evaluate whether *T. cruzi* infection activates β-catenin in BMM, the kinetics of expression of β-catenin (mRNA and protein) and its translocation to the nucleus were evaluated in *in vitro-*infected BMM at different times pi. At very short times after the infection (5 min), a significant increase in the transcription of the gene coding for β-catenin (*Ctnnb1*) was detected (Figure 3A), while the protein expression and translocation to the nucleus began at 2 h pi and reached the maximum between 8 and 12 h pi (Figures 3A,B). Moreover, the upregulation of Wnt/β-catenin pathway-target genes transcription, such as *Wisp1, Axin1*, and *Ccnd1*, correlates with β-catenin accumulation and nucleus translocation (Figure 3C). In addition, accumulation of β-catenin was observed in PM from B6-infected mice obtained at 24 h pi (Figure 3D). Taken together, our results showed that *in vitro* and *in vivo T. cruzi* infection was associated with significant activation of the genes and proteins involved in the canonical Wnt signaling pathway in macrophages.

**Figure 4:**
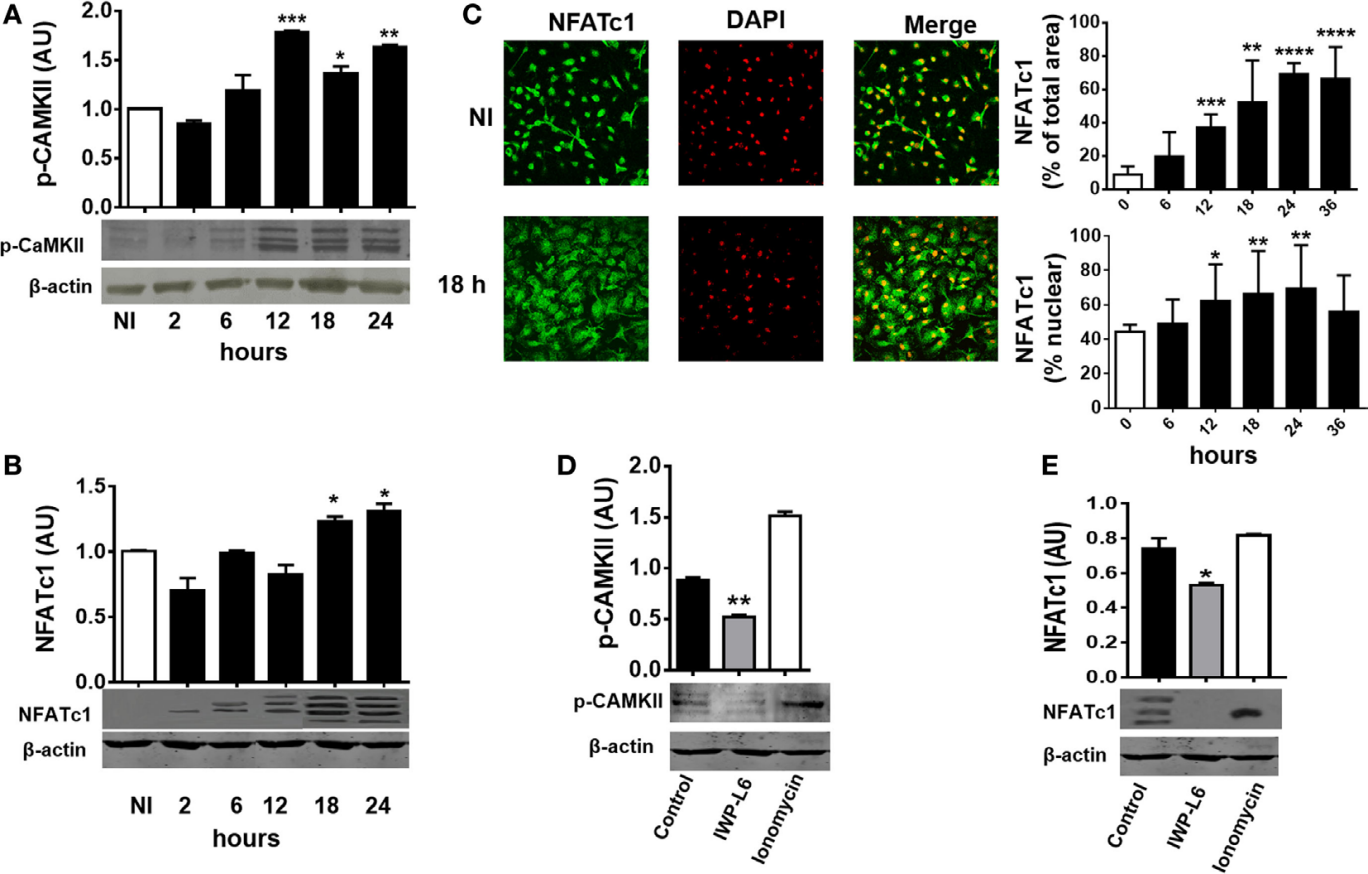
*Trypanosoma cruzi* infection induces late Wnt/Ca^+2^ pathway activation in macrophages. Bone marrow-derived macrophages were *in vitro* infected with trypomastigotes (Tps) of *T. cruzi* or left uninfected (NI) and then evaluated for calcium/calmodulin-dependent kinase II (CaMKII) phosphorylated at Thr286 expression and NFATc1 expression and localization at different times post-infection (pi). Phosphorylated CAMKII (p-CAMKII) expression **(A)** and NFATc1 expression **(B)** were assessed by Western blot and normalized to β-actin. **(C)** Expression and localization of β-catenin by immunofluorescence and confocal microscopy. A representative field for each group is shown (1,200×). Nuclear staining was detected with 4′,6-diamidino-2-phenylindone (DAPI), and the levels of expression of NFATc1 (% of total area) and the threshold Mander’s colocalization (% nuclear) coefficients calculated using FIJI/ImageJ program as described in Section “Materials and Methods.” Green, NFATc1; red, DAPI. **(D,E)** Bone marrow-derived macrophages (BMM) were treated for 24 h with the PORCN inhibitor (IWP-L6) or left untreated (control), infected with *T. cruzi* Tps and assayed for p-CaMKII and NFATc1 expression at 24 h pi. Ionomycin-activated BMM (20 min, 1 µM) were used as a positive control. In panels **(A,B,D,E)**, a representative Western blot and the ratio of protein expression to β-actin are shown. The results are expressed as the average of three independent experiments ± SEM. Abbreviation: AU, arbitrary units (*P < 0.05; **P < 0.01; ***P < 0.001; and ****P < 0.0001).

Next, we tested whether *T. cruzi* infection is also able to activate Wnt/Ca^+2^ pathway by analyzing the expression of CaMKII phosphorylated at Thr286 [phosphorylated CAMKII (p-CAMKII)] and the activation and nuclear translocation of NFATc1. As shown in Figure 4A, *T. cruzi* infection led to an increase in the phosphorylation of CaMKII at Thr286 from 12 h pi. We also detected an induced NFATc1 activation after 18 h pi as measured by faster migrating, dephosphorylated, active NFATc1 bands on Western blot (arrows; Figure 4B) and the increased expression and nuclear translocation of NFATc1 detected by immunofluorescence (Figure 4C). Interestingly, the upregulation of p-CaMKII and activation of NFATc1 were dependent on the secretion of Wnt proteins; since the use of IWP-L6, a porcupine inhibitor that is the *O*-acetyltransferase membrane involved in the palmitoylation of Wnt proteins, a critical modification for its secretion, was able to inhibit both p-CaMKII upregulation and NFATc1 activation (Figures 4D,E). Treatment of BMM with the calcium ionophore ionomycin was used as positive control of Ca^+2^ pathway activation (46). Moreover, we were unable to detect NFATc1 upregulation or translocation to the nucleus in PM obtained at 24 h pi (Figure S1 in Supplementary Material), even though it’s upregulation was evident in PM obtained from infected mice at 18 days pi (Figure S2 in Supplementary Material).

### The Inhibition of Wnt/β-Catenin Signaling Pathway Limits the Replication of *T. cruzi* in Macrophages

**Figure 5:**
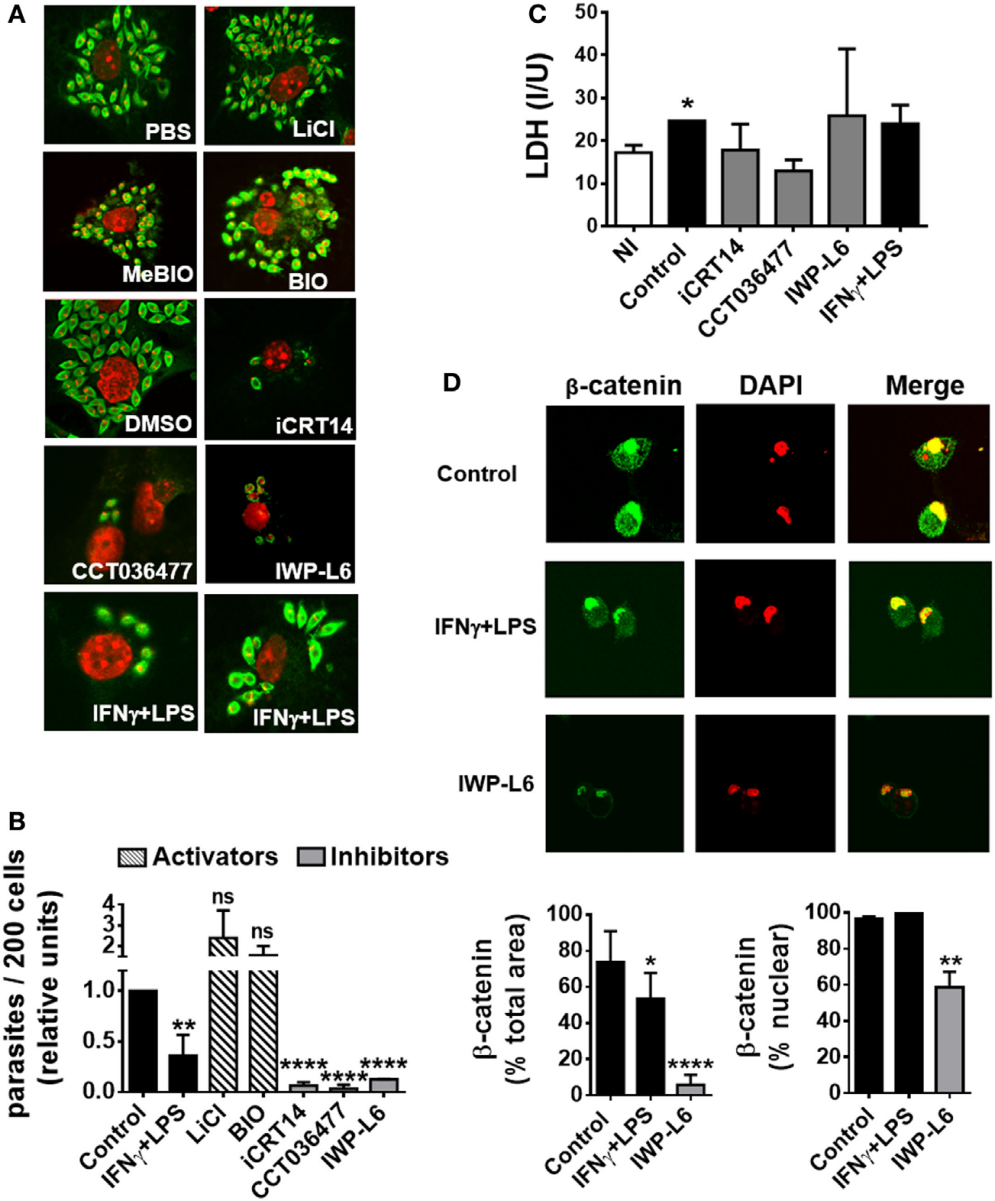
The activation of Wnt signaling pathway promotes the replication of *Trypanosoma cruzi* in macrophages. Bone marrow-derived macrophages (BMM) were treated for 24 h with β-catenin activators (LiCl or BIO), specific β-catenin transcriptional inhibitors (iCRT14 and CCT036477), PORCN inhibitor (IWP-L6), or IFNγ plus LPS as BMM activation control. Then, the cells were infected with trypomastigotes of *T. cruzi*, and intracellular parasites were counted by immunofluorescence assay. **(A)** A representative field for each group is shown (2,000×). PBS or DMSO was used as vehicle for LiCl or iCRT14, CCT036477, and IWP-L6, respectively. MeBIO was used as control of BIO treatment. **(B)** The number of intracellular amastigotes was determined by confocal microscopy. Bars represent relative units, calculated by dividing number of intracellular parasites (*n*/200 cells) in differentially treated cultures by number of intracellular parasites (*n*/200 cells) in the corresponding vehicle-treated cultures. **(C)** Lactate dehydrogenase (LDH) levels were determined in the culture supernatant of uninfected (NI) or differentially treated infected BMM at 24 h post-infection. **(D)** Effect of IWP-L6 treatment on β-catenin activation. Accumulation and nuclear translocation of β-catenin in untreated (control), IFNγ plus LPS- or IWP-L6-treated *T. cruzi-*infected BMM was calculated as described in Section “Materials and Methods.” A representative field for each group is shown (1,200×). Bars in panels **(B–D)** represent averages ± SEM from three **(C)** or four **(B,D)** independent experiments (*P < 0.05; **P < 0.01; ***P < 0.001; and ****P < 0.0001).

To evaluate the role of the Wnt/β-catenin pathway activation in the control of *T. cruzi* intracellular replication, BMM were pretreated with LiCl or BIO, both inhibitors of GSK-3β activity which mimics activation of Wnt/β catenin signaling, or specific inhibitors of β-catenin-responsive transcription such as iCRT14 and CCT036477 for 24 h before being infected. Then, the intracellular amastigotes were counted by IF assay, with the results shown in Figures 5A,B. Treatment of BMM with IFN-γ plus LPS was used to activate BMM to control *T. cruzi* replication as described previously (8). β-Catenin-responsive transcription blockade with iCRT14 and CCT036477 was shown to result in a strong inhibitory effect on intracellular parasite growth. Similar results were obtained by inhibiting the secretion of Wnt proteins with IWP-L6 (Figures 5A,B). The inhibition of parasite replication observed by β-catenin transcription blockade was even more significant than those observed after the activation of BMM with IFN-γ plus LPS, and these results were not due to drugs-induced cytotoxicity, as revealed by using LDH release assay to determine cell membrane integrity (Figure 5C). In addition, the treatment with IWP-L6 inhibited both β-catenin accumulation and nucleus translocation at 12 h pi (Figure 5D). Similar results were obtained when the drugs were added to the culture at the same time as the parasites (not shown). Likewise, inhibition of Wnt/β-catenin pathway or Wnt protein secretion in human monocyte-derived THP-1 macrophages and J774 cell line (macrophages derived from BALB/c mice) resulted in the suppression of intracellular parasite replication (Figure S3 in Supplementary Material). On the other hand, treatments with β-catenin activators (LiCl and BIO) were unable to significantly increase the intracellular parasite growth (Figures 5A,B).

**Figure 6:**
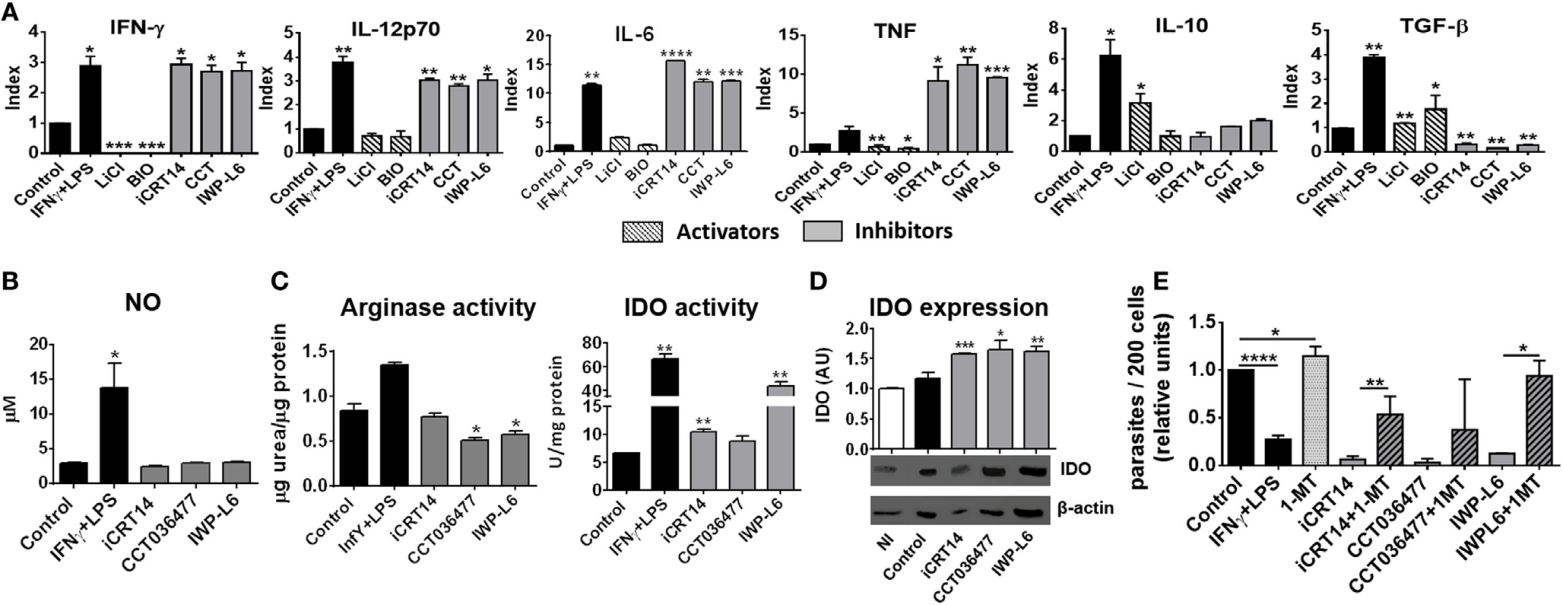
Pharmacological inhibition of Wnt pathway enhances anti-*Trypanosoma cruzi* activity of macrophages. Bone marrow-derived macrophages (BMM) were treated for 24 h with β-catenin activators (LiCl or BIO), specific β-catenin transcriptional inhibitors (iCRT14 and CCT036477), IWP-L6, 1-MT, IFNγ plus LPS or vehicle before being infected with *T. cruzi* trypomastigotes. **(A)** Cytokine levels assayed in 24 h post-infection (pi)-supernatants represented as Index obtained by dividing the cytokine release of inhibitors/activators-treated BMM for the cytokine release of the corresponding vehicle-treated BMM. The bars represent averages ± SEM from three independent experiments. **(B)** Nitrite concentration in 24 h pi-supernatant. **(C)** Arginase and indoleamine 2,3-dioxygenase (IDO) activity assayed in 24 h pi-cell **(D)** IDO expression. One representative experiment out of three performed for each condition is shown. The ratio of protein expression to β-actin is shown, and the results are expressed as the average of three independent experiments ± SEM. Abbreviation: AU, arbitrary units. Data points of nitric oxide (NO) concentrations, arginase, and IDO activity represent means ± SEM of data pooled from three cultures of the same experiment. All data are from one experiment representative of three in total. **(E)** Number of intracellular amastigotes determined by confocal microscopy at 72 h pi. The bars represent relative units, calculated as described in Figure 5B (*P < 0.05; **P < 0.01; ***P < 0.001; and ****P < 0.0001).

Then, we studied whether the inhibition of canonical Wnt signaling regulates macrophages function by modulating the secretion of pro-inflammatory and anti-inflammatory cytokines. Figure 6A shows that inhibition of Wnt/β-catenin signaling induced the secretion of the pro-inflammatory cytokines IFN-γ, IL-12, IL-6, and TNF while inhibited the production of TGF-β (Figure 6A; Table S1 in Supplementary Material), an effect that correlates with the control of parasite replication observed in iCRT14-, CCT036477-, or IWP-L6-treated BMM (Figures 5A,B). In addition, canonical signaling activation using LiCl or BIO induced a slightly upregulation of TGF-β while only LiCl increased IL-10 production by *T. cruzi*-infected BMM (Figure 6A).

Nitric oxide production is counteracted by the expression of arginase, an enzyme that competes with iNOS for l-arginine that leads to l-ornithine and urea production (47). *T. cruzi* antigens can upregulate arginase activity with this type of activation profile associated with the ability to promote the intracellular growth of *T. cruzi* (48). Interestingly, although the pharmacological inhibition of Wnt/β-catenin pathway did not affect NO production (Figure 6B), the treatments with CCT036477 and IWP-L6 significantly downregulated arginase activity in infected BMM (Figure 6C).

Because IDO activity is also critical for the control of *T. cruzi* amastigote growth in macrophages (7–9), we analyzed the effect of the inhibition of Wnt/β-catenin signaling on the activity and expression of IDO and their role in the intracellular parasite replication control. The inhibition of both β-catenin-responsive gene transcription as well as Wnt secretion induced upregulation of IDO expression and activity in infected BMM (Figures 6C,D). Remarkably, the inhibitory effect of intracellular amastigote growth induced by pharmacological inhibitors of the Wnt/β-catenin pathway was reversed when IDO activity was blocked using 1-MT (Figure 6E). In addition, as it was previously demonstrated, 1-MT exacerbated the intracellular parasite replication in untreated BMM (Figure 6E). Taken together, these results demonstrated that pharmacological inhibition of Wnt/β-catenin pathway activates macrophages to fight against *T. cruzi*.

### *In Vivo* Inhibition of Wnt Signaling Improves the Resistance to *T. cruzi* Infection

**Figure 7:**
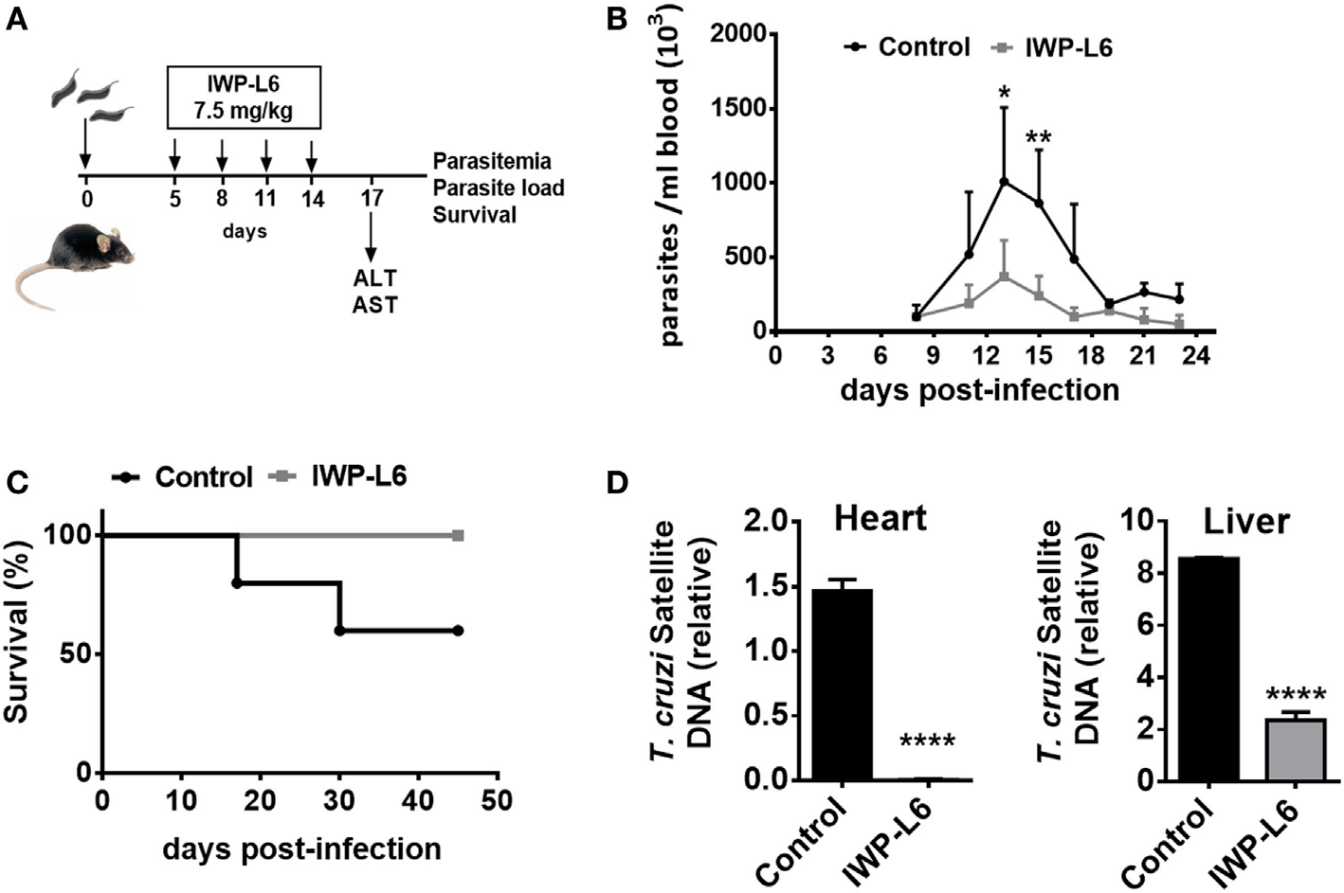
*In vivo* inhibition of Wnt signaling improves the resistance to *Trypanosoma cruzi* infection. **(A)** B6 mice infected with 5,000 *T. cruzi* trypomastigotes were treated with IWP-L6. Vehicle-treated mice were used as control. **(B)** Parasitemia. Results are means ± SD of 5–6 animals/group and are representative of three independent experiments. **(C)** Survival rate. Data are representative of one of three independent experiments. **(D)** Relative amount of *T. cruzi* satellite DNA in heart and liver from 180-day *T. cruzi-*infected IWP-L6 and control mice. Murine GAPDH was used for normalization. Data are shown as mean ± SD of triplicates (control, *n* = 4; IWP-L6, *n* = 6) mice per group (*P < 0.05; **P < 0.001; and ****P < 0.0001).

Next, we evaluated whether the inhibition of Wnt proteins secretion *in vivo* would result in the control of the parasite load. Five days after *T. cruzi* infection with a lethal dose 50 (DL50) of Tps, the mice were treated every 3 days with IWP-L6 (Figure 7A). The dose of IWP-L6 used has been previously assayed to examine the effect of Wnt proteins in an experimental model of cancer (49). As expected, *in vivo* IWP-L6 treatment could maintain inhibited both Wnt/β-catenin as well as Wnt/Ca^+2^ signaling pathways until 18 days pi, as denoted by the lack of β-catenin or NFATc1 accumulation in PM from treated versus control mice (Figure S2 in Supplementary Material). Mice infected with *T. cruzi* and injected with vehicle presented high levels of parasitemia, causing death of ~50% of mice between 18 and 30 days pi (Figure 7B). By contrast, although only four doses of IWP-L6 were given, 100% of treated mice survived to acute infection and displayed lower parasitemia than control mice during the acute phase of the infection (Figure 7C). In addition, IWP-L6-treated mice showed significantly lower parasite load in liver and heart than control mice during the chronic phase of the disease (Figure 7D).

## Discussion

Given that during the acute phase of *T. cruzi* infection macrophages can act as host cells for the parasites as well as effector cells in the early anti-parasitic immune response, the targeting of specific signaling pathways in macrophages could modulate its response to restrict parasite replication and instruct an appropriate adaptive immune response. In recent years, it has become apparent that Wnt signaling pathway, known for its essential participation in embryonic development and tissue homeostasis, exerts immunomodulatory functions during inflammation and infection.

In this study, we have demonstrated that early after the recognition of *T. cruzi* by innate immune receptors in BMM, β-catenin was activated and Wnt3a, Wnt5a, some Fzd receptors as well as target genes of Wnt/β-catenin pathway as *Axin1, Wisp1*, and *Ccnd1* were upregulated. Subsequently to canonical pathway activation, Wnt/Ca^+2^ pathway was activated; since we have demonstrated an Wnt proteins-dependent upregulated of p-CaMKII-Thr286 and activated NFATc1 expression after infection. Many studies have demonstrated that *T. cruzi* utilize the host Ca^+2^ signaling to establish the infection (50) and several mechanisms have been proposed to explain the intracellular Ca^+2^ influx that occurs during *T. cruzi* infection (51). In addition, Kayama et al. (52) have reported that NFATc1 is activated in response to *T. cruzi* infection in a TLR-independent manner, but the critical molecules and signaling pathways that lead to NFATc1 activation during *T. cruzi* infection have not yet been identified. Wnt3a and Wnt5a are more commonly associated with canonical and non-canonical Wnt signaling, respectively (19). However, Wnt5a can also activate discrete β-catenin signaling (53, 54), and recent reports have suggested that the activity of Wnt ligands and their binding to Fzd receptors depend on the cellular context; therefore, Wnt and Fzd proteins cannot be rigorously subdivided according to the pathways they induce (55). Thus, considering that we found that *T. cruzi* infection-induced β-catenin, p-CaMKII-Thr286, and activated NFATc1 upregulation were suppressed by IWP-L6 treatment, these results suggest that both Wnt3a and Wnt5a proteins, and others Wnt proteins not evaluated in this paper, could be the responsible for the activation and maintenance of both canonical and non-canonical signaling pathways during *T. cruzi* infection which allows the parasite to spread in the host.

Resistance to *T. cruzi* infection has been associated with the capacity of NK cells and T lymphocytes to generate IFN-γ which can, in turn, activate macrophages to kill the obligate intracellular amastigote form of *T. cruzi*. The trypanocidal activity of pro-inflammatory cytokines-activated macrophages is mediated at least by the upregulation of the enzymes iNOS and IDO which lead to the production of NO and kynurenines, respectively (5–9, 56). On the other hand, susceptibility to infection is associated with the production of IL-10 and transforming growth factor beta (TGF-β) (5, 56). Here, we have demonstrated that while the activation of Wnt/β-catenin pathway did not promote the intracellular parasite replication, the treatments of macrophages with specific inhibitors of β-catenin transcriptional activity or the inhibition of Wnt proteins secretion were able to inhibit the parasite replication by modifying macrophages activity. Inhibition of Wnt signaling pathway enhanced production of the pro-inflammatory cytokines IFN-γ, IL-12, TNF and IL-6 and suppressed production of TGF-β, results that are in agreement with previous reports showing that the activation of the canonical pathway in macrophages and DC controls the inflammatory response (28). In addition, these treatments induced downregulation of arginase activity, an enzyme that counteracts iNOS activity, but failed to upregulate iNOS activity, suggesting that the resulting treated-macrophages do not fully fit in the classically activated/inflammatory macrophage phenotype. Interestingly, and despite the fact that there is a close relationship between the activation of the Wnt/β-catenin pathway and the induction of IDO and *vice versa* (27, 57, 58), in our experimental model the inhibition of β-catenin-induced transcription or Wnt proteins secretion upregulated IDO expression and activity. In addition, IDO activity proved to be critical for the control of *T. cruzi* replication in BMM, as denoted by the recovery of *T. cruzi* replication observed in cultures where the inhibitors were combined with 1-MT. Expression of IDO and activation of β-catenin within macrophages and DC under tolerogenic conditions are particularly important mechanisms that limits inflammation within the gastrointestinal tract and tumor cell microenvironment (28, 59). The IDO promoter has been shown to exhibit LEF-1 binding sites, and kynurenine and quinolinic acid, produced by IDO activity, can activate the Wnt/β-catenin pathway (60, 61). However, as IDO gene expression is induced not only in tolerogenic conditions but also by IFNs and TNF during inflammatory conditions (62, 63), in our experimental settings the upregulation of IDO could be induced by the milieu of pro-inflammatory cytokines generated in inhibitors-treated *T. cruzi*-infected macrophages. Thus, our results suggest that the anti-*T. cruzi* activity of inhibitor-treated macrophages is due to the production of pro-inflammatory cytokines-inducible antimicrobial molecules, with IDO being one of the most important.

In summary, in this study, we have revealed a new signaling pathway that is activated by the interaction between protozoan parasites and host innate immunity. In this context, it is well founded that *T. cruzi* infection activates a plethora of signaling pathways that ultimately regulate the immune mediators to determine the modulation of a defined set of effector functions in macrophages and thus establishes a conceptual framework for the development of novel therapeutics.

## Ethics Statement

All animal experiments were approved by and conducted in accordance with guidelines of the Animal Care and Use Committee of the Facultad de Ciencias Químicas, Universidad Nacional de Córdoba (Approval Number HCD 831/15).

## Author Contributions

XV, LF, and CM designed the experiments. XV, LA, LF, and CI performed the experiments. XV, LF, LC, CS, and CM analyzed the data. XV, LC, and CM wrote the manuscript.

## Conflict of Interest Statement

The authors declare that the research was conducted in the absence of any commercial or financial relationships that could be construed as a potential conflict of interest.
