# Supplementary material for: Trypanosoma cruzi Exploits Wnt Signaling Pathway to Promote Its Intracellular Replication in Macrophages
Source: Front Immunol. 2018 Apr 23;9:859. doi: 10.3389/fimmu.2018.00859 (PMC5930390; doi:10.3389/fimmu.2018.00859)
Supplement: Supplementary file 1 [file presentation_1.PDF]

Supplementary Material

Trypanosoma cruzi Exploits Wnt Signaling Pathway to Promote Their Intracellular Replication in Macrophages

Ximena Volpini[1], Laura F. Ambrosio[1], Laura Fozzatti[1], Constanza Insfran[1], Cintia C. Stempin, Laura Cervi[1] and Claudia Cristina Motran[1]*.

*** Correspondence:** Corresponding Author: cmotran@fcq.unc.edu.ar

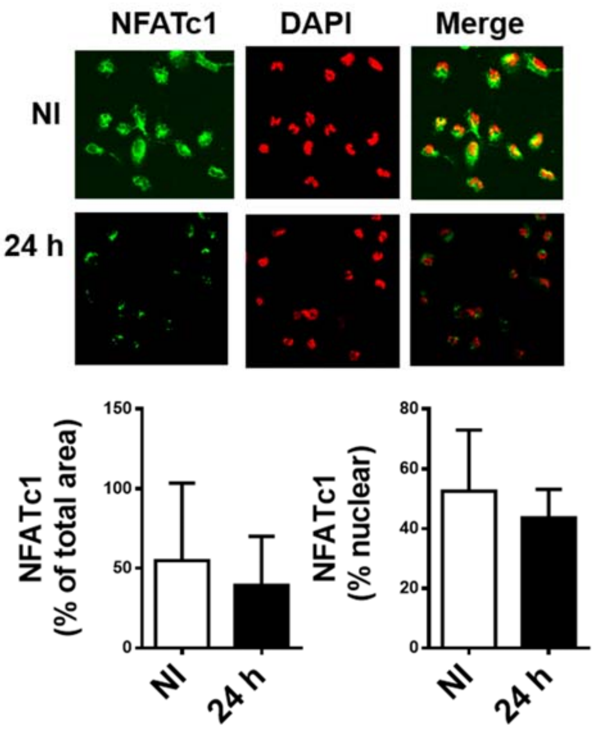

Supplementary Figure 1. NFATc1 Expression and Nuclear Translocation in Peritoneal Macrophages from 24 h-infected Mice. NFATc1 expression and localization by confocal microscopy at 24 h pi. Upper panel: a representative field for each group is shown (1200X). Nuclear staining was detected with DAPI and the levels of expression of NFATc1 (% of total area) and the threshold Mander´s colocalization (% nuclear) coefficients calculated using FIJI/ImageJ program as described in Material and Methods. Green, NFATc1; red, DAPI.

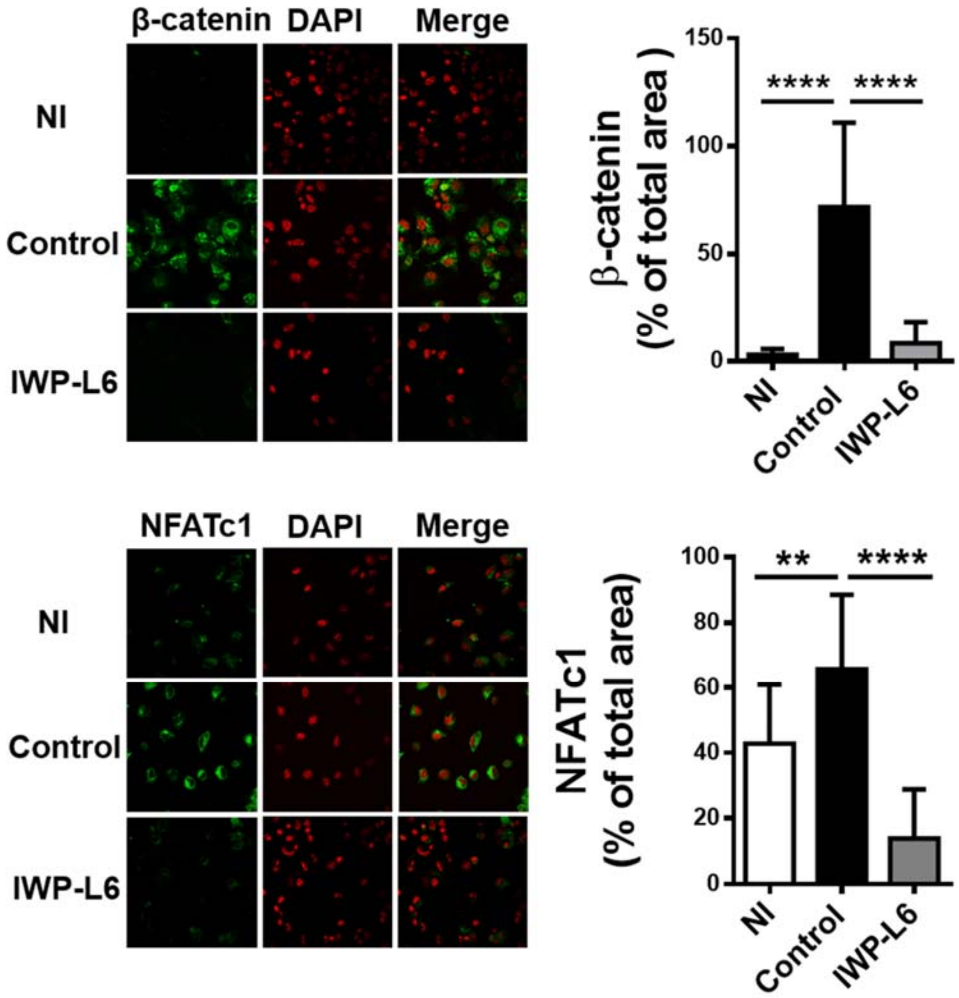

Supplementary Figure 2. β-catenin and NFATc1 Expression and Nuclear Translocation in Peritoneal Macrophages from 18 days-infected Mice. B6 mice infected with 5000 *T. cruzi* Tps were treated with IWP-L6 as described in Fig. 8A. After 18 days pi, peritoneal Mo were obtained and the expression and localization of β-catenin and NFATc1 evaluated by confocal microscopy. A representative field for each group (NI, non-infected; Control, vehicle-treated infected; IWP-L6, IWP-l6-treated infected) is shown (1200X). Nuclear staining was detected with DAPI and the levels of expression of β-catenin and NFATc1 (% of total area) and the threshold Mander´s colocalization (% nuclear) coefficients calculated using FIJI/ImageJ program as described in Material and Methods. Green, β-catenin or NFATc1; red, DAPI.

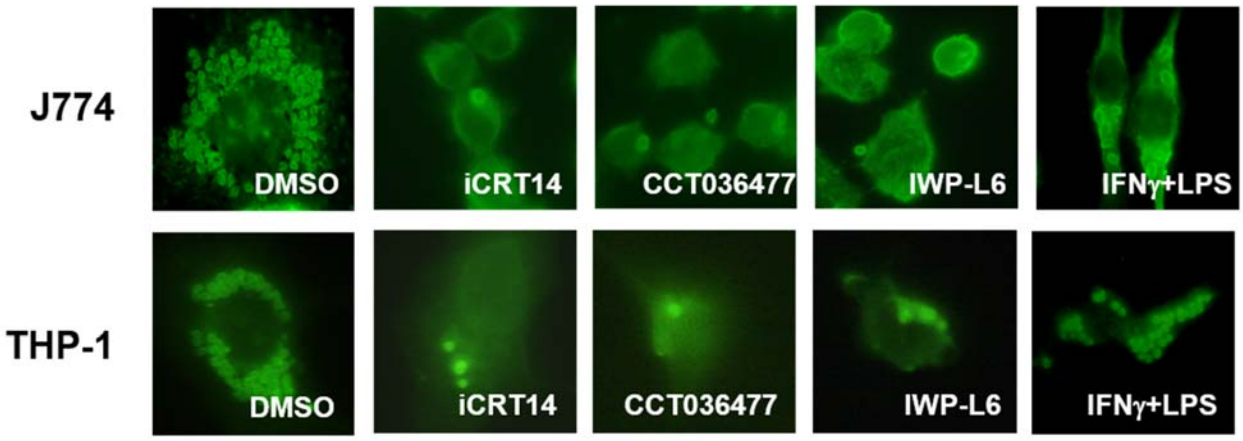

Supplementary Figure 3. The Activation of Wnt/β-catenin Signaling Pathway Promotes the Replication of *T.cruzi* in Macrophages. J774 or THP-1 Mo were treated for 24 h with specific β-catenin transcriptional inhibitors (iCRT14 and CCT036477), PORCN inhibitor (IWP-L6) or IFNγ plus LPS. Then, the cells were infected with Tps of *T. cruzi* for 24 h and intracellular parasites were evidenced by immunofluorescence assay at 72 h pi. A representative field for each group is shown (2000X). PBS or DMSO were used as controls for LiCl or iCRT14, CCT036477 and IWP-L6 respectively.

Supplementary Table 1. Summary of the mean and standard deviations of cytokine concentrations in culture supernatants obtained from differentially treated macrophages at 24 h post-infection.

Cytokine (pg/mL)	PBS	LiCl	MeBIO	BIO	DMSO	iCRT14	CCT036477	IWP-L6
IFN-γ	1118±104	52±4	1135±35	47±3	1180±113	3038±19	2425±3	2662±8
IL-12	280±14	291±10	353±9	355±9	252±17	578±20	532±21	578±14
IL-6	20±4	47±5	20±7	19±1	21±2	310±1	238±15	241±5
TNF	238±9	212±22	238±5	214±27	170±22	1893±117	1730±123	1483±17
IL-10	54±2	159±3	64±8	61±2	92±1	117±2	129±3	138±3
TGF-β	1055±91	1567±10	909±16	1106±58	1475±78	459±5	486±6	732±9
